# Supplementary material for: Metabolic modelling reveals increased autonomy and antagonism in type 2 diabetic gut microbiota
Source: Mol Syst Biol. 2025 Apr 22;21(6):720–31. doi: 10.1038/s44320-025-00100-w (PMC12130202; doi:10.1038/s44320-025-00100-w)
Supplement: Supplementary file 1 — Appendix [file 44320_2025_100_MOESM1_ESM.pdf]

The appendix for: Metabolic modelling reveals increased autonomy and antagonism in type 2 diabetic gut microbiota

Table of contents:

Appendix Table S1	Alpha diversity measures vs. diabetes, fasting glucose, and medications anti-diabetes
Appendix Figure S1	The ecological interaction rations between diabetic and non-diabetic microbial communities, Mann-Whitney U test
Appendix Figure S2	Metabolic fluxes associated with diabetes
Appendix Table S2	metabolic groups
Appendix Table S3	Statistical test results
Appendix Figure S3	Histogram distributions explaining the metadata
Appendix Table S4	dada2 package functions used for the 16s sequence preparation
Appendix Table S5	The standardised proportion of each metabolite from its metabolic group, estimated depending on the reference diet.
Appendix Table S6	Ecological interaction types and categories
Appendix Table S7	The pathways and their reactions that are positively associated with blood glucose

Appendix Table S1: Alpha diversity measures vs. diabetes, fasting glucose, and medications anti-diabetes.

* Significantly increased alpha diversity with the observation, LM: Linear models with confounders. All P-values are adjusted for false discovery rates (FDR correction)

	Diabetes		Higher fasting glucose	Anti-diabetic medications	
	Mann-Whitney, FDR P-values	LM, FDR P-values	LM, FDR P-values	Mann-Whitney, FDR P-values	LM, FDR P-values
Shannon	5.4×10^{-6} *	0.051	0.08	0.44	0.37
Chao1	2.1×10^{-4} *	0.14	0.08	0.45	0.62
Species richness	3.7×10^{-5} *	0.003 *	0.32	0.49	0.37
Simpson’s	1.1×10^{-3} *	0.5	0.09	0.45	0.37

Appendix Figure S1: The ecological interaction ratios between diabetic and non-diabetic microbial communities, Mann-Whitney U test.

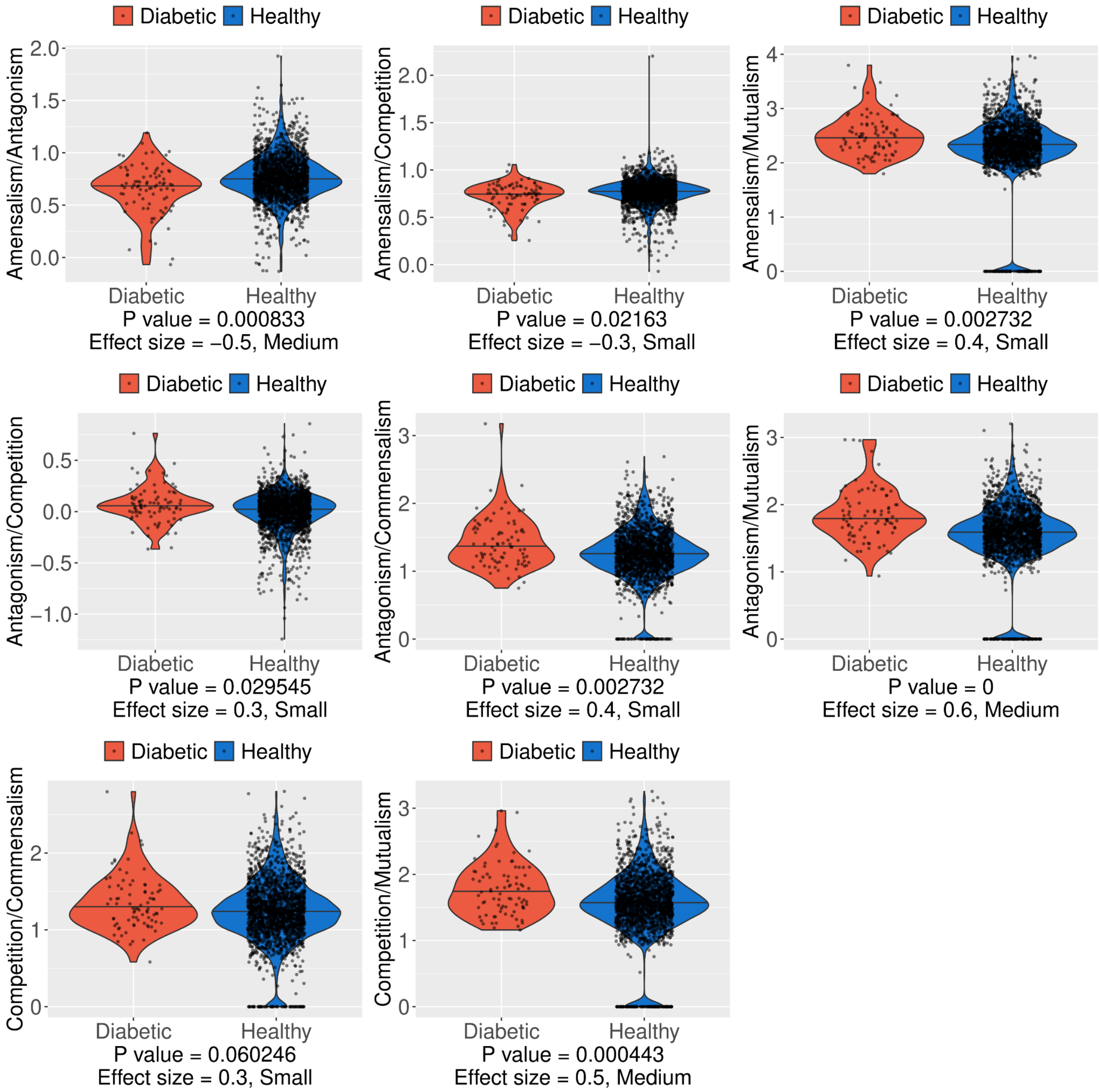

Each dot represents the corresponding ecological interaction ratio in one microbiome. The Violin shapes depict the distribution of those ratios among all communities, and the black horizontal lines in the middle of the violins represent the median value for each of the distributions. The black lines in the middle of each box plot represent the median value, the bottom and top box edges represent the first and third quartals respectively, the dots above and below the boxes represent potential outliers and the last points at the bottom and at the top of each box represent the minimum and maximum values respectively. The number of samples for the red boxes is the number of diabetic patients (n1 = 81), and for the blue boxes is the number of non-diabetic individuals (n2 = 1,785).

Appendix Figure S2: Metabolic fluxes associated with diabetes, to the left increased in healthy and to the right increased in diabetic.

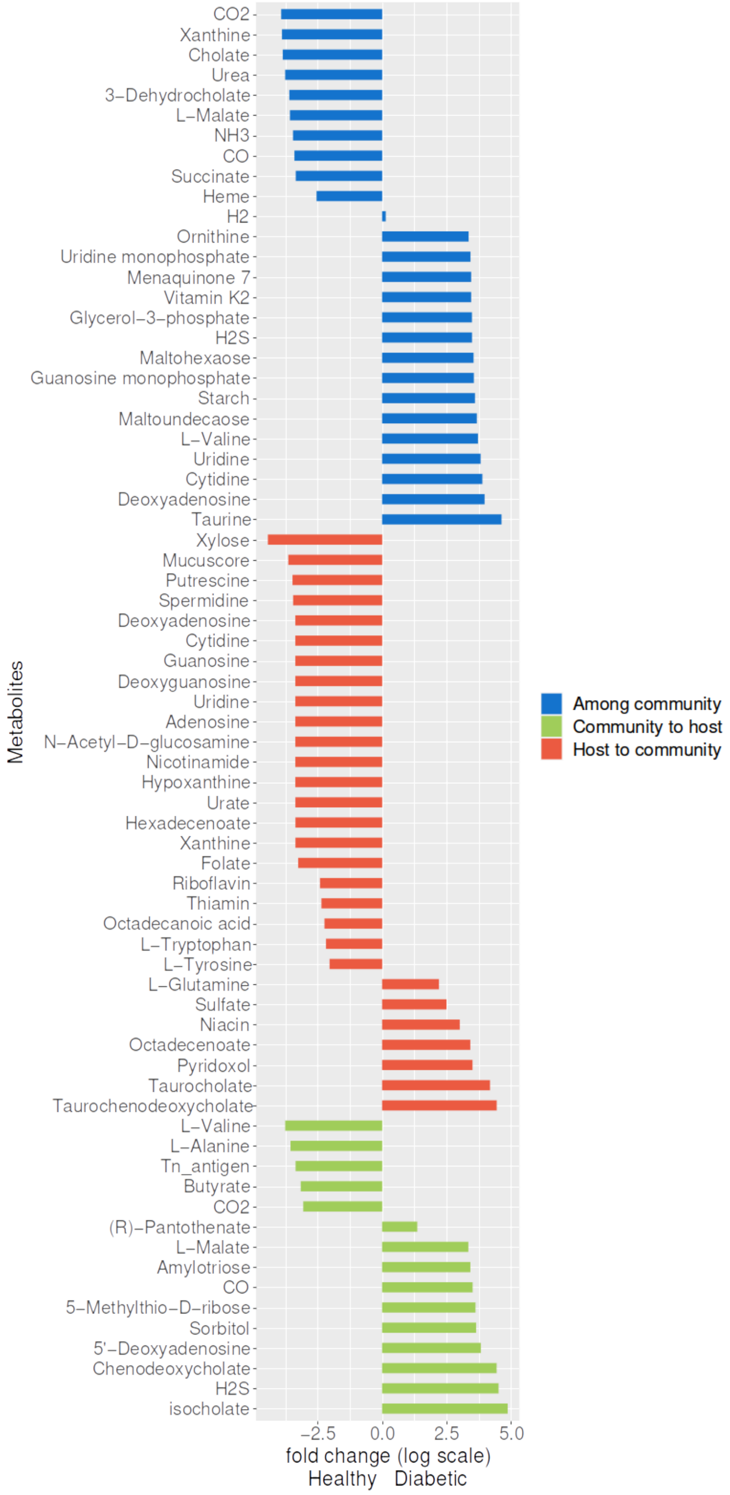

Appendix Table S2:

Metabolite ID	Metabolite Name	Metabolic Group
cpd00156	L-Valine	Amino Acids
cpd00239	H2S	Inorganic Compounds
cpd00048_o	Sulfate_host	Inorganic Compounds
cpd15269_o	Octadecenoate_host	Fatty Acids
cpd00033_o	Glycine_host	Amino Acids
cpd00030_o	Mn2+_host	Electrolytes
cpd00034_o	Zn2+_host	Electrolytes
cpd00058_o	Cu2+_host	Electrolytes
cpd00063_o	Ca2+_host	Electrolytes
cpd00099_o	Cl-_host	Electrolytes
cpd00107_o	L-Leucine_host	Amino Acids
cpd00205_o	K+_host	Electrolytes
cpd00239_o	H2S_host	Inorganic Compounds
cpd00254_o	Mg_host	Electrolytes
cpd00091	Uridine monophosphate	Nucleotides
cpd00149_o	Co2+_host	Inorganic Compounds
cpd00053_o	L-Glutamine_host	Amino Acids
cpd00126	Guanosine monophosphate	Nucleotides
cpd01663_o	Chenodeoxycholate_host	Bile Acids
cpd00305_o	Thiamin_host	Vitamins
cpd03246_o	Taurochenodeoxycholate_host	Bile Acids
cpd01981_o	5-Methylthio-D-ribose_host	Carbohydrates
cpd90004	Starch	Carbohydrates
cpd00359	Indol	Organic Compounds
cpd00010	CoA	Energy Rich Molecules
cpd00448	D-Glyceraldehyde	Intermediates
cpd90007	Maltohexaose	Organic Compounds
cpd00009_o	Phosphate_host	Energy Rich Molecules
cpd00218	Niacin	Vitamins
cpd00132_o	L-Asparagine_host	Amino Acids

cpd03847_o	Myristic acid_host	Fatty Acids
cpd00046	Cytidine monophosphate	Nucleotides
cpd00220_o	Riboflavin_host	Vitamins
cpd00204_o	CO_host	Inorganic Compounds
cpd00141_o	Propionate_host	Fatty Acids
cpd00092	Uracil	Nucleotides
cpd00029_o	Acetate_host	Fatty Acids
cpd00322_o	L-Isoleucine_host	Amino Acids
cpd00069_o	L-Tyrosine_host	Amino Acids
cpd00367	Cytidine	Nucleotides
cpd00051_o	L-Arginine_host	Amino Acids
cpd90006	Maltoundecaose	Organic Compounds
cpd01741_o	Dodecanoate_host	Fatty Acids
cpd03047_o	Taurocholate_host	Bile Acids
cpd00263_o	Pyridoxol_host	Organic Compounds
cpd01080_o	Octadecanoic acid_host	Organic Compounds
cpd00003	NAD	Nucleotides
cpd00644_o	(R)-Pantothenate_host	Organic Compounds
cpd00036_o	Succinate_host	Intermediates
cpd00080	Glycerol-3-phosphate	Intermediates
cpd00119_o	L-Histidine_host	Amino Acids
cpd00064	Ornithine	Amino Acids
cpd00130_o	L-Malate_host	Decarboxylic acids
cpd00065_o	L-Tryptophan_host	Amino Acids
cpd11451	Vitamin K2	Vitamins
cpd11606	Menaquinone 7	Vitamins
cpd00076_o	Sucrose_host	Carbohydrates
cpd00179	Maltose	Carbohydrates
cpd00060_o	L-Methionine_host	Amino Acids
cpd00161_o	L-Threonine_host	Amino Acids
cpd00100	Glycerol	Alcohols
cpd00129_o	L-Proline_host	Amino Acids
cpd00159_o	L-Lactate_host	Carbohydrates
cpd00067_o	H+_host	Electrolytes

cpd00106	Fumarate	Intermediates
cpd00363_o	Ethanol_host	Alcohols
cpd11640_o	H2_host	Inorganic Compounds
cpd00182	Adenosine	Nucleotides
cpd00232_o	Neu5Ac_host	Decarboxylic acids
cpd00438	Deoxyadenosine	Nucleotides
cpd00218_o	Niacin_host	Vitamins
cpd00249	Uridine	Nucleotides
cpd00300_o	Urate_host	Organic Compounds
cpd00047_o	Formate_host	Intermediates
cpd00023_o	L-Glutamate_host	Amino Acids
cpd00226	Hypoxanthine	Purine bases
cpd00794	D-Trehalose	Organic Compounds
cpd00039_o	L-Lysine_host	Amino Acids
cpd00013_o	NH3_host	Inorganic Compounds
cpd00011_o	CO2_host	Inorganic Compounds
cpd90003_o	Starch_host	Carbohydrates
cpd01262_o	Amylotriose_host	Carbohydrates
cpd10516_o	fe3_host	Electrolytes
cpd00066_o	L-Phenylalanine_host	Amino Acids
cpd00229_o	Glycolaldehyde_host	Organic Compounds
cpd00027	D-Glucose	Carbohydrates
cpd00064_o	Ornithine_host	Amino Acids
cpd00133	Nicotinamide	Vitamins
cpd00307	Cytosine	Amino Acids
cpd00084_o	L-Cysteine_host	Amino Acids
cpd00184_o	Thymidine_host	Nucleotides
cpd00211_o	Butyrate_host	Fatty Acids
cpd90005	Maltooctaose	Carbohydrates
cpd00832_o	N-Acetyl-D-chondrosamine _host	Organic Compounds
cpd00082_o	D-Fructose_host	Carbohydrates
cpd00210	Taurine	Amino Acids
cpd90008	Maltotridecaose	Carbohydrates

cpd23430_o	isocholate_host	Organic Compounds
cpd00054_o	L-Serine_host	Amino Acids
cpd00082	D-Fructose	Carbohydrates
cpd00028	Heme	Intermediates
cpd00588_o	Sorbitol_host	Alcohols
cpd00001	H2O	Inorganic Compounds
cpd00047	Formate	Intermediates
cpd00066	L-Phenylalanine	Amino Acids
cpd00363	Ethanol	Alcohols
cpd00012	PPi	Intermediates
cpd01861_o	1,2-Propanediol_host	Organic Compounds
cpd00311	Guanosine	Nucleotides
cpd00309	Xanthine	Purine bases
cpd00071	Acetaldehyde	Intermediates
cpd00055	Formaldehyde	Intermediates
cpd00035	L-Alanine	Amino Acids
cpd00117	D-Alanine	Amino Acids
cpd03091_o	5'-Deoxyadenosine_host	Nucleotides
cpd00154_o	Xylose_host	Carbohydrates
cpd01618_o	1,3-Propanediol_host	Alcohols
cpd00033	Glycine	Amino Acids
cpd10515_o	Fe2+_host	Electrolytes
cpd00011	CO2	Inorganic Compounds
cpd00013	NH3	Inorganic Compounds
cpd00035_o	L-Alanine_host	Amino Acids
cpd00036	Succinate	Intermediates
cpd00073	Urea	Organic Compounds
cpd00118_o	Putrescine_host	Intermediates
cpd00122_o	N-Acetyl-D-glucosamine_host	Carbohydrates
cpd00130	L-Malate	Decarboxylic acids
cpd00133_o	Nicotinamide_host	Vitamins
cpd00156_o	L-Valine_host	Amino Acids
cpd00182_o	Adenosine_host	Nucleotides

cpd00204	CO	Inorganic Compounds
cpd00226_o	Hypoxanthine_host	Purine bases
cpd00249_o	Uridine_host	Nucleotides
cpd00264_o	Spermidine_host	Polyamines
cpd00277_o	Deoxyguanosine_host	Nucleotides
cpd00309_o	Xanthine_host	Purine bases
cpd00311_o	Guanosine_host	Nucleotides
cpd00367_o	Cytidine_host	Nucleotides
cpd00393_o	Folate_host	Vitamins
cpd00438_o	Deoxyadenosine_host	Nucleotides
cpd00526	Cholate	Bile Acids
cpd00654_o	Deoxycytidine_host	Nucleotides
cpd02992_o	Mucuscore_host	Mucus compounds
cpd11640	H2	Inorganic Compounds
cpd15237_o	Hexadecenoate_host	Fatty Acids
cpd21520_o	Tn_antigen_host	Carbohydrates
cpd23431	3-Dehydrocholate	Bile Acids
cpd00654	Deoxycytidine	Nucleotides

Appendix Table S3:

**Abundances vs. diabetes**				
**Species**	**Test**	**Increased In**	**Fdr P.value**	**Significance**
Ruminococcus gnavus ATCC 29149	Mann–Whitney U test	Diabetis	<0.0001	**
Granulicatella adiacens ATCC 49175	Mann–Whitney U test	Diabetis	<0.0001	**
Clostridium celatum DSM 1785	Mann–Whitney U test	Healthy	<0.0001	**
Clostridium clostridioforme CM201	Mann–Whitney U test	Diabetis	<0.0001	**
Streptococcus australis ATCC 700641	Mann–Whitney U test	Diabetis	0.00089	**
Streptococcus mitis NCTC 12261	Mann–Whitney U test	Diabetis	0.001	*
Ruminococcus torques ATCC 27756	Mann–Whitney U test	Diabetis	0.0047	*
Streptococcus parasanguinis ATCC 903	Mann–Whitney U test	Diabetis	0.013	*

Lachnospiraceae bacterium sp 5 1 63FAA	Mann–Whitney U test	Healthy	0.02	*
Bacteroides vulgatus ATCC 8482	Mann–Whitney U test	Diabetis	0.023	*
Eubacterium rectale ATCC 33656	Mann–Whitney U test	Healthy	0.032	*
**Ecological Interactions vs. diabetes**				
**Ecological Interaction Ratio**	**Test**	**Increased In**	**Fdr P.value**	**Significance**
Amensalism/Antagonism	Mann–Whitney U test	Healthy	0.00083	**
Amensalism/Competition	Mann–Whitney U test	Healthy	0.022	*
Amensalism/Commensalism	Mann–Whitney U test	Equal	0.48	*
Amensalism/Neutralism	Mann–Whitney U test	Equal	0.85	*
Amensalism/Mutualism	Mann–Whitney U test	Diabetis	0.0027	*
Antagonism/Competition	Mann–Whitney U test	Diabetis	0.03	*
Antagonism/Commensalism	Mann–Whitney U test	Diabetis	0.0027	*
Antagonism/Neutralism	Mann–Whitney U test	Equal	0.13	*
Antagonism/Mutualism	Mann–Whitney U test	Diabetis	<0.0001	*
Competition/Commensalism	Mann–Whitney U test	Equal	0.06	*
Competition/Neutralism	Mann–Whitney U test	Equal	0.49	*
Competition/Mutualism	Mann–Whitney U test	Diabetis	0.00044	**
Commensalism/Neutralism	Mann–Whitney U test	Equal	0.49	*
Commensalism/Mutualism	Mann–Whitney U test	Diabetis	0.0068	*
Neutralism/Mutualism	Mann–Whitney U test	Diabetis	0.04	*
**Ecological interactions vs. blood glucose**				
**Ecological Interaction Ratio**	**Test**	**Estimate**	**Fdr P.value**	**Significance**
Amensalism/Antagonism	Linear Model	-3.18	0.057	*
Amensalism/Competition	Linear Model	-4.95	0.057	*
Amensalism/Commensalism	Linear Model	1.21	0.095	*
Amensalism/Neutralism	Linear Model	-0.2	0.6	*
Amensalism/Mutualism	Linear Model	0.9	0.095	*
Antagonism/Competition	Linear Model	1.34	0.54	*
Antagonism/Commensalism	Linear Model	2.55	0.0097	*
Antagonism/Neutralism	Linear Model	-0.073	0.86	*
Antagonism/Mutualism	Linear Model	1.94	0.0097	*
Competition/Commensalism	Linear Model	2.05	0.016	*
Competition/Neutralism	Linear Model	-0.069	0.86	*
Competition/Mutualism	Linear Model	1.62	0.016	*

Commensalism/Neutralism	Linear Model	-0.88	0.26	*
Commensalism/Mutualism	Linear Model	0.74	0.55	*
Neutralism/Mutualism	Linear Model	1.15	0.12	*
**Metabolic fluxes vs. blood glucose**				
**Metabolites**	**Flux direction**	**LM estimate**	**Fdr P.value**	**Significance**
CO2	Community to Host	-0.03	0.0065	*
Butyrate	Community to Host	-0.1	0.0065	*
Succinate	Community to Host	0.02	0.0213	*
H+	Community to Host	0.02	0.0393	*
chenodeoxycholate	Community to Host	16.19	0.0452	*
Folate	Diet to Community	-22.19	0.0025	*
D-Glucose	Diet to Community	0.65	0.0087	*
Spermidine	Diet to Community	-26.23	0.0283	*
Adenosine	Diet to Community	-7.38	0.0338	*
Uridine	Diet to Community	-7.38	0.0338	*
Deoxyguanosine	Diet to Community	-6.46	0.0338	*
urate	Diet to Community	-0.96	0.0338	*
Guanosine	Diet to Community	-6.46	0.0338	*
Cytidine	Diet to Community	-6.46	0.0338	*
Deoxyadenosine	Diet to Community	-7.38	0.0338	*
HYXN	Diet to Community	-3.05	0.0347	*
N-Acetyl-D-glucosamine	Diet to Community	-0.25	0.0347	*
LACT	Diet to Community	49.02	0.0302	*
L-Fucose	Diet to Community	681.38	0.0307	*
L-Glutamine	Diet to Community	7.83	0.0454	*
Folate	Within Community	21.1	0.0025	*
GMP	Within Community	0.89	0.0087	*
mql7	Within Community	0.09	0.0087	*
Menaquinone 7	Within Community	0.09	0.0087	*
Ornithine	Within Community	1.79	0.0139	*
Deoxyadenosine	Within Community	2.73	0.0213	*
Putrescine	Within Community	28.61	0.0322	*
L-Lysine	Within Community	3.82	0.0338	*
1,2-Propanediol	Within Community	7.09	0.0349	*
Spermidine	Within Community	10.81	0.0486	*
D-Lactate	Within Community	16.84	0.0347	*
Galactose	Within Community	23.85	0.0454	*

Metabolic fluxes vs. Diabetes				
**Metabolites**	**Flux direction**	**Increased In**	**Fdr P.value**	**Significance**
Folate	Diet to Community	Healthy	<0.0001	**
L-Valine	Within Community	Diabetis	<0.0001	**
Spermidine	Diet to Community	Healthy	<0.0001	**
Adenosine	Diet to Community	Healthy	<0.0001	**
Uridine	Diet to Community	Healthy	<0.0001	**
Deoxyguanosine	Diet to Community	Healthy	<0.0001	**
Guanosine	Diet to Community	Healthy	<0.0001	**
Cytidine	Diet to Community	Healthy	<0.0001	**
Deoxyadenosine	Diet to Community	Healthy	<0.0001	**
cholate	Within Community	Healthy	<0.0001	**
N-Acetyl-D-glucosamine	Diet to Community	Healthy	<0.0001	**
Nicotinamide	Diet to Community	Healthy	<0.0001	**
HYXN	Diet to Community	Healthy	<0.0001	**
Deoxycytidine	Diet to Community	Healthy	<0.0001	**
GMP	Within Community	Diabetis	<0.0001	**
urate	Diet to Community	Healthy	<0.0001	**
XAN	Diet to Community	Healthy	<0.0001	**
UMP	Within Community	Diabetis	<0.0001	**
H2S	Within Community	Diabetis	<0.0001	**
L-Alanine	Community to Host	Healthy	<0.0001	**
chenodeoxycholate	Community to Host	Diabetis	<0.0001	**
L-Malate	Community to Host	Healthy	<0.0001	**
Xylose	Diet to Community	Healthy	<0.0001	**
octadecenoate	Diet to Community	Diabetis	<0.0001	**
5-Methylthio-D-ribose	Community to Host	Diabetis	<0.0001	**
L-Valine	Community to Host	Healthy	<0.0001	**
taurocholate	Diet to Community	Diabetis	0.0001	**
L-Tyrosine	Diet to Community	Healthy	0.0002	**
Sulfate	Diet to Community	Diabetis	0.0003	**
taurochenodeoxycholate	Diet to Community	Diabetis	0.0003	**
mucuscore	Diet to Community	Healthy	0.0004	**
Urea	Within Community	Healthy	0.0005	**
Succinate	Within Community	Healthy	0.0006	**
starch (n=19, 3xalpha1-6, 15xalpha1-4)	Within Community	Diabetis	0.0008	**
Deoxyadenosine	Within Community	Diabetis	0.001	*

Riboflavin	Diet to Community	Healthy	0.001	*
H2S	Community to Host	Diabetis	0.001	*
NH3	Within Community	Healthy	0.0014	*
Thiamin	Diet to Community	Healthy	0.0015	*
3-dehydrocholate	Within Community	Healthy	0.0029	*
Maltoundecaose	Within Community	Diabetis	0.0039	*
Maltohexaose	Within Community	Diabetis	0.0045	*
Tn_antigen	Community to Host	Healthy	0.0051	*
Putrescine	Diet to Community	Healthy	0.0073	*
L-Malate	Within Community	Healthy	0.0083	*
CO2	Community to Host	Healthy	0.0085	*
L-Tryptophan	Diet to Community	Healthy	0.0085	*
Ornithine	Within Community	Diabetis	0.0117	*
L-Glutamine	Diet to Community	Diabetis	0.0125	*
Heme	Within Community	Healthy	0.0126	*
Uridine	Within Community	Diabetis	0.0171	*
Cytidine	Within Community	Diabetis	0.0201	*
CO2	Within Community	Healthy	0.0203	*
Amylotriose	Community to Host	Diabetis	0.0259	*
CO	Within Community	Healthy	0.0259	*
mql7	Within Community	Diabetis	0.0259	*
Menaquinone 7	Within Community	Diabetis	0.0259	*
XAN	Within Community	Healthy	0.0306	*
H2	Within Community	Diabetis	0.0306	*
Niacin	Diet to Community	Diabetis	0.0345	*
Glycerol-3-phosphate	Within Community	Diabetis	0.0369	*
PAN	Community to Host	Diabetis	0.0369	*
isocholate	Community to Host	Diabetis	0.0377	*
Sorbitol	Community to Host	Diabetis	0.0378	*
Butyrate	Community to Host	Healthy	0.038	*
CO	Community to Host	Diabetis	0.0403	*
hexadecenoate	Diet to Community	Healthy	0.0403	*
ocdca	Diet to Community	Healthy	0.0431	*
Taurine	Within Community	Diabetis	0.0469	*
pydxn	Diet to Community	Diabetis	0.0491	*
5'-Deoxyadenosine	Community to Host	Diabetis	0.0498	*

Subsystems Enriched with Metabolic Reactions Associated with Blood Glucose				
**Subsystem**	**Number of Reactions Associated with Blood Glucose**	**Estimate**	**Fdr P.value**	**Significance**
Glycolysis I (From Glucose 6-Phosphate)	21	3.496	0.0068	*
Glycolysis Iii (From Glucose)	27	2.519	0.009	*
Bifidobacterium Shunt	28	2.614	0.009	*
Bifidoshunt2	27	2.553	0.009	*
Glycolysis Ii (From Fructose 6-Phosphate)	18	3.307	0.009	*
L-Histidine Biosynthesis	11	5.893	0.009	*
Glycolysis V (Pyrococcus)	14	3.753	0.0137	*
Utp And Ctp Dn Biosynthesis	11	4.505	0.0158	*
Pyrimidine Deoxyribonucleotide Phosphorylation	12	4.179	0.0158	*
Adenosine Deoxyribonucleotides Dn Biosynthesis Ii	12	3.98	0.0162	*
Guanosine Deoxyribonucleotides Dn Biosynthesis Ii	12	3.98	0.0162	*
Cmp Phosphorylation	10	4.64	0.0186	*
Gluconeogenesis I	12	3.633	0.0254	*
Sucrose Degradation Ii (Sucrose Synthase)	9	4.472	0.0365	*
Pyrimidine Deoxyribonucleotides Dn Biosynthesis Ii	14	2.955	0.0403	*
Guanosine Ribonucleotides Dn Biosynthesis	10	3.662	0.0405	*
Peptidoglycansyn-Pwy2	8	4.635	0.0405	*
Methylerythritol Phosphate Pathway I	7	5.405	0.0405	*
Pwy-8073	6	6.947	0.0405	*
Lipid Iva Biosynthesis	6	6.947	0.0405	*

Appendix Figure S3: Histogram distributions explaining the metadata

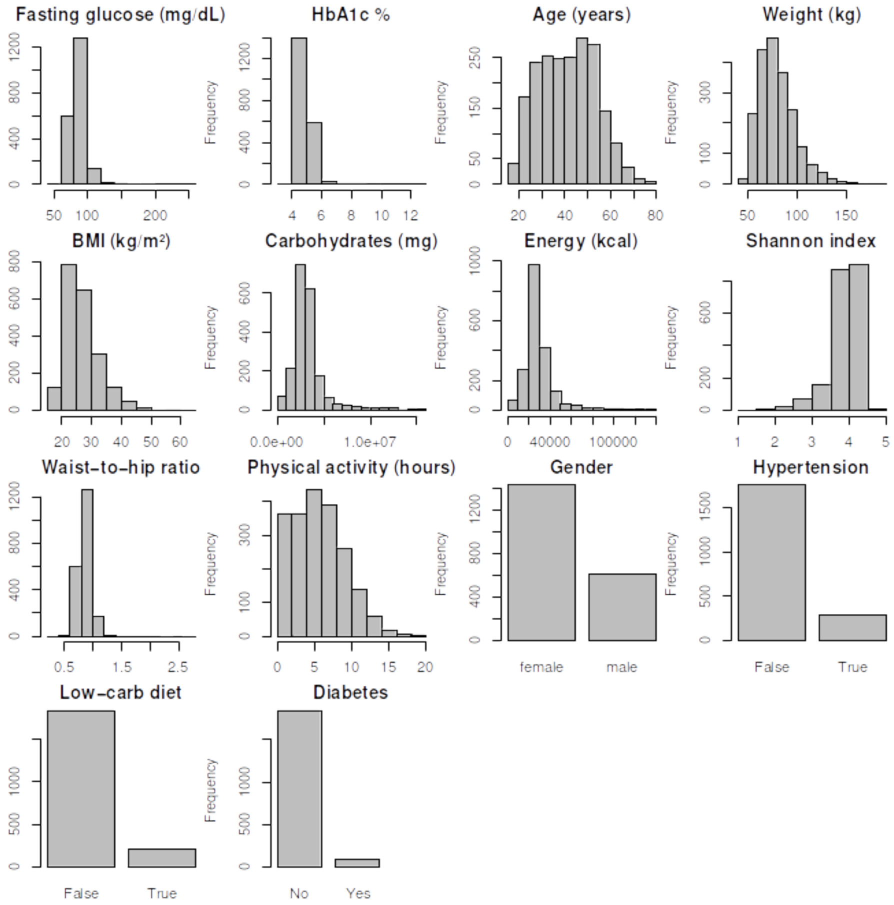

Appendix Table S4: dada2 package functions used for the 16s sequence preparation

learnErrors	Used for estimating error rates (kmer based).
plotErrors	Provides a graphical representation of the error rate.
filterAndTrim	Refines sequencing data by filtering out low-quality reads and trimming sequences.
derepFastq	Removes duplicate sequences.
dada	Used for denoising sequencing data and resolving unique variants.
mergePairs	Aligns and merges paired-end sequencing reads.

Appendix Table S5:

**Metabolite ID**	**Ratio from its group**
cpd00035	0.04624039423
cpd00051	0.06094241002
cpd00041	0.1005489044
cpd00084	0.01213306225
cpd00023	0.2294063924
cpd00033	0.03887113993
cpd00119	0.02231877476
cpd00322	0.0447459331
cpd00107	0.07569698669
cpd00039	0.05984404047
cpd00060	0.01735873353
cpd00066	0.04119122065
cpd00129	0.0698044616
cpd00054	0.0463125599
cpd00161	0.03943520857
cpd00065	0.01094401869
cpd00069	0.03266765952
cpd00156	0.0515380993
cpd01107	0.008075596777
cpd01741	0.02285409452
cpd03847	0.03784772666
cpd15298	0.003842447073

cpd15622	0.003537653962
cpd00214	0.19908331
cpd15237	0.01697905434
cpd15609	0.003243410595
cpd01080	0.07030063116
cpd15269	0.2450190117
cpd01122	0.1555769353
cpd03850	0.05957870555
cpd15016	0.003002994243
cpd03848	0.003677268907
cpd02077	0.0008904873436
cpd00188	0.002675479612
cpd05231	0.01184288725
cpd16342	0.0003101115653
cpd03852	0.01142862527
cpd05235	0.0003121860306
cpd05238	0.0005507457163
cpd00211	0.008142363127
cpd03846	0.005014214953
cpd00160	0.004992390457
cpd00100	0.1212216679
cpd00314	0.007721613484
cpd00588	0.01497030761
cpd00306	4.89E-05
cpd27312	0.06932920764
cpd00208	0.109339614
cpd00179	0.02562247606
cpd00076	0.2504267601
cpd00082	0.1067551515
cpd00108	0.003341956235
cpd00027	0.09857296045
cpd00155	0.06761723016
cpd01262	0.02052496633
cpd90003	0.2257288453
cpd00063	0.0004088868804

cpd00099	0.002989009369
cpd00058	0.00130042675
cpd10516	0.0003330332455
cpd00534	7.64E-05
cpd00205	0.001601412324
cpd00254	0.0002109935189
cpd00030	0.00203798097
cpd00971	0.002221854442
cpd00009	0.0008069917943
cpd00048	0.0005538194271
cpd00034	0.007567678467
cpd01420	0.002834355687
cpd00365	0.008556764553
cpd00305	0.0006859072328
cpd00635	6.54E-06
cpd00220	0.001002844703
cpd00218	0.01065559537
cpd00644	0.003270371324
cpd00263	0.0009187246551
cpd00104	0.0001449022404
cpd00393	0.0001853191302
cpd03224	7.16E-05
cpd01628	0.01488997715
cpd01401	0.0003043273471
cpd00363	0.01280039828
cpd00001	0.5818053385

Appendix Table S6: Ecological interaction types and categories

**Species A**	**Species B**	**Ecological interaction**	**Interaction Category**
Benefit	Benefit	Mutualism	Collaborative
No change	Benefit	Commensalism	Collaborative

Benefit	Harm	Antagonism	Exploitative
No change	No change	Neutralism	Neutral
No change	Harm	Amensalism	Exploitative
Harm	Harm	Competition	Exploitative

Appendix Table S7: The pathways and their reactions that are positively associated with blood glucose

**Glycolysis I (from glucose 6−phosphate), ten reactions:** 1) glucose-6-phosphate isomerase, 2) 6-phosphofructokinase, 3) fructose-bisphosphate aldolase, 4) triose-phosphate isomerase, 5) glyceraldehyde-3-phosphate dehydrogenase, 6) phosphoglycerate kinase, 7) 2,3-bisphosphoglycerate-independent phosphoglycerate mutase, 8) phosphoglycerate mutase, 9) enolase, 10) pyruvate kinase.
**Gluconeogenesis I, nine reactions;** 1) malate dehydrogenase, 2) 2-phosphoenolpyruvate carboxykinase, 3) enolase, 4) 2,3-bisphosphoglycerate-independent phosphoglycerate mutase, 5) phosphoglycerate kinase, 6) glyceraldehyde-3-phosphate dehydrogenase, 7) triose-phosphate isomerase, 8) fructose-bisphosphate aldolase, 9) Missing reaction: fructose-1,6-bisphosphatase, 10) glucose-6-phosphate isomerase
**Calvin−Benson−Bassham cycle, four reactions:** 1)Phosphoglycerate kinase, 2)triosephosphate isomerase, 3)sedoheptulose-1,7-bisphosphate aldolase, 4)D-fructose, 6)phosphate: D-glyceraldehyde-3-phosphate glycolaldehydetransferase
**Bifidobacterium shunt, ten reactions;** 1)ATP: D-glucose 6-phosphotransferase, 2)Glucose-6 phosphate isomerase, 3)ATP: acetate phosphotransferase, 4)sedoheptulose-7-phosphate, 5)D-Fructose 6-phosphate, 6)xylulose-5-phosphate phosphoketolase, 7)D-glyceraldehyde-3-phosphate, 8)ATP:3-phospho-D-glycerate 1-phosphotransferase, 9)2-Phospho-D-glycerate 2,3-phosphomutase, 10)ATP: pyruvate 2-O-phosphotransferase.
**Mixed acid fermentation, six reactions:** 1) pyruvate kinase, 2) phosphate acetyltransferase, 3) acetate kinase, 4) malate dehydrogenase, 5) fumarase, 6) fumarate reductase
**Sucrose degradation IV (sucrose phosphorylase), three reactions:** 1) phosphoglucomutase, 2) fructokinase, 3) glucose-6 phosphate isomerase
